# Interferon and interferon-stimulated genes in HBV treatment

**DOI:** 10.3389/fimmu.2022.1034968

**Published:** 2022-12-01

**Authors:** Qirong Li, Baozhen Sun, Yue Zhuo, Ziping Jiang, Rong Li, Chao Lin, Ye Jin, Yongjian Gao, Dongxu Wang

**Affiliations:** ^1^ Department of Gastrointestinal Colorectal and Anal Surgery, China-Japan Union Hospital of Jilin University, Changchun, China; ^2^ Laboratory Animal Center, College of Animal Science, Jilin University, Changchun, China; ^3^ Department of Hepatobiliary and Pancreas Surgery, China-Japan Union Hospital of Jilin University, Changchun, China; ^4^ School of Acupuncture-Moxi bustion and Tuina, Changchun University of Chinese Medicine, Changchun, China; ^5^ Department of Hand and Foot Surgery, The First Hospital of Jilin University, Changchun, China; ^6^ School of Grain Science and Technology, Jilin Business and Technology College, Changchun, China; ^7^ School of Pharmacy, Changchun University of Chinese Medicine, Changchun, China

**Keywords:** HBV, IFN, ISGs, IFN-α, Peg-IFN-α

## Abstract

Human hepatitis B virus (HBV) is a small enveloped DNA virus with a complex life cycle. It is the causative agent of acute and chronic hepatitis. HBV can resist immune system responses and often causes persistent chronic infections. HBV is the leading cause of liver cancer and cirrhosis. Interferons (IFNs) are cytokines with antiviral, immunomodulatory, and antitumor properties. IFNs are glycoproteins with a strong antiviral activity that plays an important role in adaptive and innate immune responses. They are classified into three categories (type I, II, and III) based on the structure of their cell-surface receptors. As an effective drug for controlling chronic viral infections, Type I IFNs are approved to be clinically used for the treatment of HBV infection. The therapeutic effect of interferon will be enhanced when combined with other drugs. IFNs play a biological function by inducing the expression of hundreds of IFN-stimulated genes (ISGs) in the host cells, which are responsible for the inhibiting of HBV replication, transcription, and other important processes. Animal models of HBV, such as chimpanzees, are also important tools for studying IFN treatment and ISG regulation. In the present review, we summarized the recent progress in IFN-HBV treatment and focused on its mechanism through the interaction between HBV and ISGs.

## Introduction

Hepatitis B virus (HBV) infection and its related diseases is an important medical problem in China and all over the world. In addition to causing chronic hepatitis B (CHB), it is a major cause of advanced liver disease and hepatocellular carcinoma (HCC) (1). HBV is a non-cytopathic DNA virus, belonging to the hepatophilic DNA virus family (2). Chronic HBV infection can cause persistent low-grade hepatic inflammation in patients, accompanied by transient episodes of high hepatic inflammation and the development of fibrotic processes, which results in liver fibrosis, cirrhosis, and ultimately decompensated liver disease or HCC in 25–40% of patients (3). CHB is characterized by the persistence of free covalently closed circular DNA (cccDNA) of the HBV genome as a stable miniature chromosome in the nucleus of infected hepatocytes (4). After treatment discontinuation or loss of immune defense, HBV cccDNA multiples in hepatocytes and can reactivate viral replication to produce an intact virus (5). Therefore, complete elimination of cccDNA from infected hepatocytes is important to achieve complete elimination of HBV. However, the presently available therapies can only control HBV infection or replication and cannot cure it completely. Previous studies have divided HBV cures into “functional” and “complete” (6). Functional cure refers to serum clearance of hepatitis B surface antigen (HBsAg), which is sometimes accompanied by serum DNA and continuously transcribed inactive cccDNA. Complete cure refers to the complete elimination of cccDNA (6).

HBV infection is generally controlled by reverse transcriptase inhibitors (nucleosides or nucleotide analogs [NAs]) and interferon (IFN) therapy (7). Presently, antiviral drugs approved for CHB treatment can be divided into two major groups. One is pegylated IFN-α (PEG-IFN-α), which inhibits viral replication in about 25% of patients (8). The other is the new generation of NAs that have high antiviral potency and resistance barriers and produce strong viral suppression in many patients (9). IFNs are a group of cytokines first discovered and explored in 1957. It is a key regulator of the immune response process against various viruses and cancers and also one of the first lines of defense for host cells against viruses (10). The following three types of IFNs are found: I (α, β, 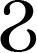, κ, and ε), II (γ), and III (λ). IFN complexes can activate the Janus-activated kinase (JAK)-signal transducer and the activator of the transcription (STAT) pathway, which leads to the expression of IFN-stimulated genes (ISGs). These genes can further regulate viral replication and immune response as downstream effectors (11). The proteins encoded by ISGs inhibit the proliferation of viruses by inhibiting their transcription, translation, and replication, which promotes the degradation of viral nucleic acid, and changes the cellular lipid metabolism level (12).

Studies have shown that ISG expression is associated with HBV infection and treatment (13). IFNs can regulate almost 10% of genes in the human genome. The proteins encoded by ISGs can individually or collectively play a role in inducing the intrinsic antiviral proliferation activity of cells and activating adaptive immunity for antiviral defense (14). In this review, we mainly focus on the mechanism underlying the treatment of IFNs, emphasizing the regulation of ISGs. Elucidating the regulatory mechanism underlying ISGs is helpful to understand their future impact better on antiviral therapy and pave the way for research of long-term HBV control therapy and the identification of new therapeutic targets.

## HBV

CHB is prevalent in Africa, Asia, and parts of Central and Eastern Europe. Nearly 1 million people die every year due to complications of persistent HBV infection, cirrhosis, and HCC, with 250 million people affected by CHB globally (15). The present research has reported the gene expression and replication mechanisms underlying the HBV life cycle. Viral and host determinants influence whether the virus can successfully infect (16). Studies have reported that HBV naturally infects humans, chimpanzees, and some primates to a lesser extent. The parenchymal cells in the liver are the only sites where HBV can multiply (17).

### HBV pathogenesis and clinical diagnosis

HBV can be transmitted through infected bodily fluids such as blood and semen, which can be caused immune-mediated liver disease (7). HBV does not directly damage cells. The inflammation and necrosis of liver tissue are mainly due to the host’s recognition of invading antigens and the activation of its own immune system, which targets and destroys infected hepatocytes. Liver injury caused by excessive immune activation can further contribute to liver fibrotic disease and HCC during chronic HBV infection (18). HBV is highly effective in invading recognition by the innate immune system owing to its unique replication strategies, such as the use of capped and polyglandulated transcripts similar to host-derived mRNAs or the restriction of RNA/DNA genomes produced by replication to nucleocapsid particles in the cytoplasm (19). The HBV genotypes can be classified based on their genome sequences from A to J with many subtypes (20). The pathogenicity, virulence, clinical outcome, and response of HBV to type I IFN treatment are associated with its genotype. HBV DNA levels and hepatitis B E antigen (HBeAg) seroconversion rates were lower in patients infected with HBV genotypes C or D than those with HBV genotypes A or B (21). HBeAg seroconversion rate refers to patients who no longer express HBeAg and produce anti-HBeAg antibodies (22). HBV infection can be divided into the following four stages: immune tolerance, HBeAg positive immune active, HBeAg negative immune inactive (CHB loss or low replication), and HBeAg negative immune active (23). Serological markers should be discovered to determine the disease stage. General serum markers can diagnose CHB and help in distinguishing between acute and chronic infections. Common serological tests can detect HBV surface antigen (HBS), HBeAg, HBV surface antibody (anti-HBS), hepatitis B core antibody (anti-HBC), HBV envelope antibody (anti-HBE), and HBV DNA (24).

### HBV life cycle and infection process

**Figure 1:**
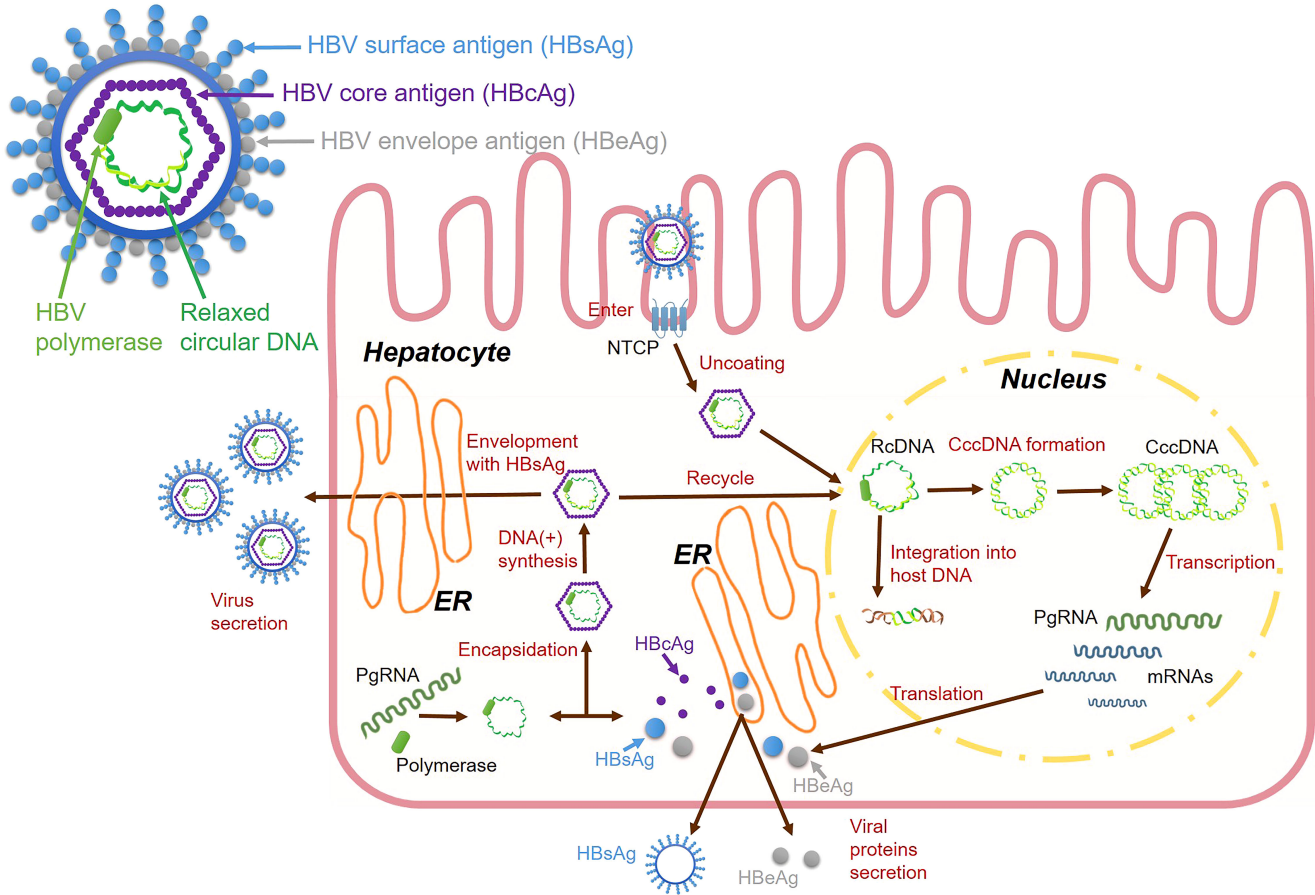
HBV virions and HBV life cycle.

During the HBV life cycle, HBV DNA is transformed into a highly stable double-stranded circular DNA structure called cccDNA, which is an important stage in the nucleus of the liver cells. During this stage, cccDNA is integrated into the host genome as a template for viral RNA transcription, and cccDNA hides in the nuclei of the liver cell nuclei and serves as a template for viral replication (25) (**Figure 1**). HBV infectious virions are enveloped nucleocapsids that selectively enter hepatocytes and deliver incomplete circular DNA genomes, which initiates multiple viral replication processes (26). The circulating virions are initially attached to heparan sulfate proteoglycans (HSPG) (27), then viral surface proteins facilitate their entry into host hepatocytes. The preS1 domain is a crucial structure for mediating large surface proteins (28). HBV can enter the hepatocytes, which is co-mediated by surface molecules called sodium taurocholate cotransport polypeptides (NTCPs) (29). After the entry of the virus, the HBV nucleocapsid containing relaxed circular DNA (rcDNA) is delivered into the nucleus, where host enzymes transform the viral genome into cccDNA (30). Human RNA polymerase II mediates cccDNA transcription to produce pregenomic RNA (pgRNA). PgRNAs are mRNAs of core proteins and polymerases that serve as templates for HBV DNA replication (31). PgRNA is reverse transcribed to form incomplete rcDNA, wherein the HBV capsid is coated with HBsAg to become mature virus particles (32). Capsid-containing rcDNAs are transported back to the nucleus to increase the cccDNA content or enter multivesicular bodies. They come into contact with viral envelope proteins and exit hepatocytes to circulate in the blood as infectious virions (33).

### HBV animal model

Establishing animal HBV infection models is important for elucidating the mechanism underlying the immune response to HBV infection, which leads to hepatitis and the progression of liver injury and repair. Establishing relevant animal models has facilitated the development of methods to control chronic HBV infection and the study of ISG regulatory pathways. Mice have good immune system characteristics and are easy to handle. However, they cannot naturally be infected with HBV. Therefore, many studies have established various HBV infection models in transgenic mice using gene editing and humanized liver technologies (34). Past studies have found that sterile alpha motif domain-containing 4A (SAMD4A) is an important anti-HBV ISG by overexpressing or knocking down ISGs in HBV transgenic mice (35). Besides, interferon alpha-inducible protein 27 (IFI27) as ISG can inhibit HBV gene expression and DNA replication in mouse models (36). Other studies have shown that the steady-state level of HBV DNA in ubiquitin specific peptidase 18 (USP18) (UBP43) deficient mice is significantly reduced (37). Moreover, some studies have used the human liver chimeric mouse model and shown that HBV/HDV infection significantly induced ISG expression (38). Chimpanzees are the only immunocompetent animals that are naturally susceptible to HBV, and they are the main animal model for studying HBV infection (39). However, their HBV-related studies were limited because of ethical issues. Other animals, such as woodchucks, are naturally infected with hepatitis viruses similar to HBV (40). Woodchucks can be infected with woodchucks hepatitis virus (WHV), and ducks can be infected with duck hepatitis virus (41). These viruses have characteristics similar to HBV infection in humans. Some studies have investigated the changes in ISG expression after HBV infection using custom woodchuck microarray platforms (42). Moreover, another HBV-like virus, woolly monkey HBV (WMHBV), can infect its natural host, woolly monkeys, and was investigated for antiviral therapies for HBV infection (43). Other smaller non-human primate models are also being developed, such as tupaias, cynomolgus monkeys, and rhesus monkeys. The development of these animal models is crucial for studying HBV infection (44) (**Table 1**). HBV has a high species specificity. However, recent advances in transgenic mice, humanized mice, and strategies to make macaques more susceptible to HBV infection are gradually improving our ability to study HBV in a more suitable *in vivo* environment (47).

**Table 1:** Animal models for HBV researches.

Animal species	Hepadna-virus	Naturally Susceptible	Experimental infection mode	Advantages and disadvantages
**Mouse** (45)	HBV	No	HBV transgenic mouse model	The HBV transgenic mouse model can be widely used for several preclinical HBV antiviral evaluations *in vivo*. However, HBV transgenic mice revealed innate immune tolerance to HBV, while no covalently closed circular cccDNA was detected.
			Humanized Chimera Mouse	The best model for studying HBV persistence is the humanized xenograft model, albeit it is limited by a high degree of immune-deficiency.
**Chimpanzee (** 46)	HBV	Yes	Can be directly infected	It can accurately simulate the pathogenesis and disease progression caused by human HBV. However, the availability constraints, high associated costs, and considerable ethical concerns have limited their use as experimental models.
**Capuchin monkey (** 34)	HBV	Yes	Can be directly infected	Capuchin monkeys are highly endangered, have limited availability, are of xenogeneic origin, and have poorly characterized immune systems.
**Rhesus macaques (** 44)	HBV	No	Exogenous expression of human NTCP on the surface of hepatocytes	It is the only available, non-endangered HBV NHP model. However, it is not susceptible to HBV infection, which has a low level of replication.
**Tupaia (** 44)	HBV	Yes	Can be directly infected	It is very sensitive to HBV, but has the genetic heterogeneity of outbred species, the overall virus titer *in vivo* is low, and the research tools and materials for this species are scarce.
**Woolly monkey (** 43)	WMHBV	Yes	Can be directly infected	The species is highly endangered and impossible to study.
**Woodchuck (** 40)	WHBV	Yes	Can be directly infected	It has long been applied as a model to explore the biology and pathogenesis of hepatophilic DNA viruses as well as to evaluate antiviral drugs. However, the viral sequence homology between WHBV and HBV is limited, and the reagents used to characterize the immune system of marmots are insufficient.
**Duck (** 41)	DHBV	Yes	Can be directly infected	DHBV can effectively replicate after infection, and infected cells can release infectious virus particles. However, the viral sequence homology between DHBV and HBV is limited, and ducks are distant from humans.

### HBV treatment with IFNs

The prophylactic vaccine for HBV is adopted in all developed countries. It is a common and crucial measure for preventing and controlling HBV (48). However, this vaccine does not affect patients with prolonged infections. Currently, treatment for these patients is limited to immunomodulators, including many direct-acting antivirals (DAAs), known as third-generation nuclear analogs (NUCs), such as entecavir, tenofovir, and tenofovir alanine or regular and pegylated type I IFNs (7). Induction of IFN expression occurs in response to viral or bacterial infection. With the development of recombinant IFNs, IFNs have been increasingly applied in HBV treatment, and have become a more popular treatment option (49).

## IFNs and PEG-IFNs

When the HBV viral load is low, it can induce a type I IFN response and stimulate HBV gene expression and replication (50). However, type I IFNs inhibits HBV replication when the viral load is high. IFN-α and IFN-γ can interfere with the synthesis of negative-strand DNA virus by inducing apolipoprotein B mRNA editing enzyme catalytic subunit 3G (APOBEC3G) expression and binding to viral DNA polymerase (51). Therefore, type I IFNs can promote or inhibit HBV infection depending on the viral expression.

IFN-α induces genes encoding intracellular or secreted proteins (ISGs) that promote immune cell activation. They have direct or indirect antiviral activity (52). Human IFN-α can reduce HBV DNA, HBeAg, and HBsAg levels in hepatocytes (53). Furthermore, IFN-α14 can be the most effective IFN subtype for inhibiting HBV cccDNA transcription and HBeAg/HBsAg production. IFN-α14 can activate IFN-α and IFN-γ signaling and induce the expression of many potent antiviral effectors, synergistically limiting HBV replication (54). The anti-HBV activity of IFN-α is regulated by a complex mode of action, which includes natural killer (NK) T cell activation (55). They decrease pgRNA and subgenomic RNA transcription in HBV cccDNA microsomes and decrease signal transducer and activator of transcription 1 (STAT1) and 2 (STAT2) transcription factor binding to active cccDNA, which collectively inhibit HBV replication (56). IFN-α can be used to treat HBV by degrading cccDNA *via APOBEC3A* activation in infected cells (56). Furthermore, IFN-α treatment significantly upregulated the expression of the host gene ubiquitin-conjugating enzyme E2 L3 (*UBE2L3*), whereas *UBE2L3* silencing increased the antiviral activity of IFN-α against HBV RNA, cccDNA, and DNA (57). IFN-α can also transfer antiviral molecules from cell to cell through exosomes, which contributes to its antiviral response to HBV in mice (58). Cross-linking IFN-α with apolipoprotein A-I produces a molecule with different antiviral and immune-stimulating activities that decrease IFN-α hematologic toxicity and have HBV therapeutic effects (59). Moreover, IFNs inhibit HBV secretion by inducing the protein Tetherin, which is the potential anti-HBV response mechanism triggered by IFNs (60).

PEG-IFN-α is added to some therapeutic agents that are pegylated by partially incorporating polyethylene into the active product. PEG-IFN-α molecules are mainly used to increase the pharmacokinetic properties of unmodified IFN-α (61). The binding of pegylated molecules to IFNs increases its half-life more than that by IFN-α alone. This reduces its rates of absorption and renal and cellular clearance. Moreover, PEG-IFN-α requires less frequent administration than IFN-α and produced more durable viral inhibitory effects in clinical trials (62). A recent study created and evaluated two pegylated IFN preparations (PEG IFN-α-2a and PEG IFN-α-2b) with different molecular sizes and structures, *in vivo* and *in vitro* properties, and half-lives (63, 64). The immunomodulatory function of PEG-IFN-α, especially NK cell activation, plays a key role in response to HBV treatment (65). Furthermore, PEG-IFN-α-2b improved the resistance of CHB patients to HBV by increasing the number of HBV-specific CD8^+^ T cells and regulating the expression of Th1 and Th2 cytokines (66). PEG-IFN-α treatment upregulates exosomal microRNAs (miRNAs) miR-193a-5p, miR-25-5p, and miR-574-5p, with exosomes secreted by macrophages transferring IFN-α-related miRNA into HBV-infected hepatocytes, which inhibits HBV replication and transcription (67).

### IFNs and PEG-IFNs clinical practice in HBV treatment

Systematic reviews and meta-analyses of the role of conventional IFN-α in patients with HBeAg-positive CHB have found that it can improve their biological, serological, and virological responses. Treatment with higher doses of IFN-α and a longer duration of continuous administration can have a better therapeutic effect; however, it can also lead to side effects and increased treatment costs (68). IFN-α is presently the first choice of antiviral therapy for children with CHB older than one year, whereas PEG-IFN-α-2a is the recommended treatment for children with CHB older than three years. The results showed that antiviral monotherapy with IFNα-2B or PEG-IFNα-2a was well tolerated and effective in CHB children compared with adults with higher HBeAg seroconversion rates and HBsAg clearance rates (69). Many studies have shown that standard IFN-α has a specific role in anti-HBV infection; however, pure IFN-α is not commonly given as a therapy in clinical trials (70). Some studies have shown that IFN-α treatment is ineffective in most patients with HBV infection possibly because HBV prevents the induction of IFN-α signaling and interferes with ISG transcription in hepatocytes by inhibiting STAT1 nuclear translocation, which results in a low IFN-α therapeutic effectiveness (71). Overall, its antiviral effects in patients with CHB are modest for unknown reasons but may include inadequate delivery to the infected liver, tolerance of infected hepatocytes to IFN-α signaling, or other mechanisms (72).

Clinical results showed that PEG-IFN-α-2b was effective in treating HBeAg-positive CHB (73). In addition, PEG-IFN-α monotherapy was effective in 298 Chinese inactive HBV carriers, with good tolerability and safety (74). Using PEG-IFN-α in treating HBeAg-positive patients with CHB could inhibit viral production to some extent in 10%–40% of patients, and the HBeAg serum conversion rate of patients was about 25%–30%. Loss of HBsAg expression was observed in approximately 5% of patients six months after treatment discontinuation (75). Treatment regimens with PEG-IFN-α should be determined based on host-related factors and viral predictive markers, such as age, alanine transaminase (ALT) levels, viral load, and HBV genotype (76). Moreover, hepatitis B core-related antigen and HBsAb levels at the end of treatment can help determine the curative effect of PEG-IFN-α-based treatment in patients with CHB (77).

### Clinical use of IFNs and PEG-IFNs combined with other drugs in HBV treatment

Combining IFN-α and PEG-IFN-α with other drugs is currently an attractive approach. The co-administration of ribavirin and IFN-α may be effective in treating viremic anti-HBE-positive patients with CHB who have not responded well to previous IFN treatment (78). Another clinical trial showed that sequential combination therapy with lamivudine and IFN-α induced a sustained virological response, including HBS seroconversion, in patients with CHB who were unresponsive to IFN-α alone. This observation suggests that this treatment regimen needs to be further evaluated in clinical trials (79). NVR3-778 is one of the core protein allosteric modulators (CpAMs), which has been shown to reactivate the host innate immune response by inducing the expression of ISGs (80, 81). Clinical studies have shown that combining PEG-IFN-α and NVR3-778 exerts a good antiviral effect *in vivo* (82). In addition, combining entecavir or tenofovir with PEG-IFN-α can reduce HBsAg levels consistently (83). Additional treatment with PEG-IFN-α results in higher serological response rates than monotherapy and may facilitate NAs discontinuation (84).

Furthermore, current regimens that may be of more interest include combining IFNs with traditional Chinese medicine (TCM) (85). Many studies have reported that TCM and related active compounds extracted from TCM have a potential anti-HBV activity, including *Salvia miltiorrhiza*, *Astragalus*, *Oxymatrine*, *Artemisinin*, and *Vogoning*. TCM preparations have better safety than IFN-α regarding dose-dependent side effects and drug resistance and are potential candidates for anti-HBV therapies (86). TCM preparations combined with IFNs considerably decreased serum HBeAg, increased serum HBV DNA clearance rates, and improved serum ALT normalization compared with IFNs alone (87). Moreover, a polysaccharide from *Radix isatidis* (*Isatis indigotica* Fortune) can exert an antiviral effect by activating the IFN-α-dependent JAK/STAT signaling pathway and increasing anti-HBV protein levels (88). Despite many IFN-related clinical trials, stronger evidence and more detailed experiments are needed to evaluate the safety and efficacy of combination therapy. In addition, more studies are needed to develop more convenient and effective IFN-α-based HBV treatment strategies.

## Interaction between HBV and ISGs

After HBV infection, the host can induce many ISGs, the core components of intracellular antiviral innate immunity (89). ISGs can regulate IFN signaling and even directly inhibit viral infection. Studies on ISG mechanisms can show how IFN-induced signaling reprograms and primes cells to enhance viral detection, achieve effective viral defense, and return cells to normal functions. In addition, some studies have shown that ISGs are related to treating HBV using IFN-α (90). In the present study, we focused on the role of ISGs in treating HBV by regulating type I IFNs.

### IFNs and ISGs

**Figure 2:**
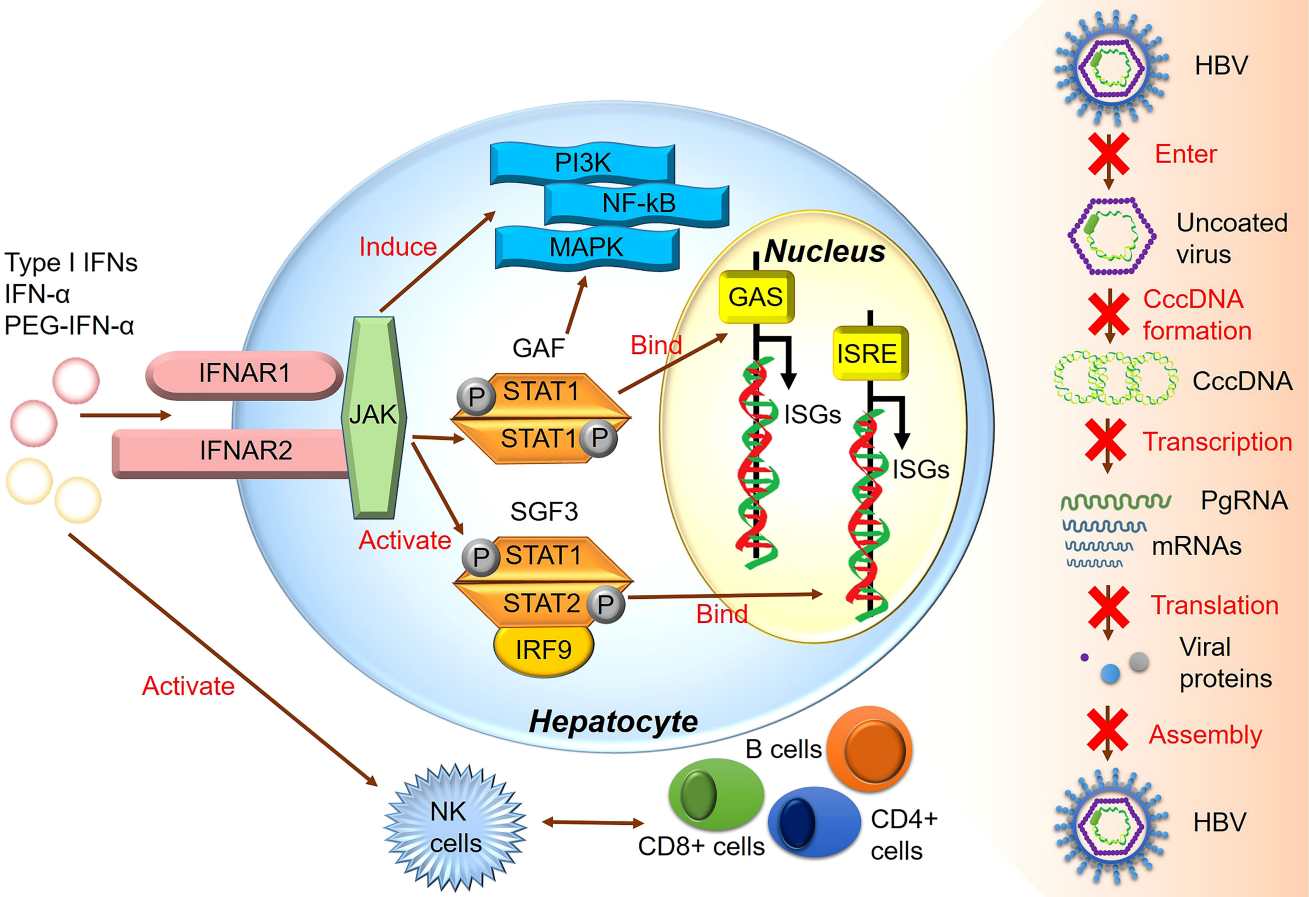
The major signaling pathway through which IFN produces its inhibitory effect on HBV.

All type I IFNs, including IFN-α and IFN-β, are regulated by the IFN-α/β receptor (IFNAR) complex, which contains two subunits, IFNAR1 and IFNAR2 (91). However, type I IFN binding to IFNAR can induce ISG expression and activate the JAK/STAT signaling pathway (92). The heterotrimeric ISG factor 3 (ISGF3) transcription factor complex comprises phosphorylated STAT1/STAT2 and interferon regulatory factor 9, and type I IFNs can activate *ISGF3* expression *via* the JAK/STAT signaling pathway (14). Activated ISGF3 binds to ISG upstream promoter regions in the nucleus in response to IFN stimulation (**Figure 2**). Furthermore, studies have shown that the increased interaction between STAT1 methylation and STAT1- protein inhibitor of activated STAT-1 is involved in IFN-α HBV antagonism, and the antiviral effect of IFN-α can be enhanced by increasing the expression of methylated STAT1 and S-adenosyl methionine (93). In addition, the unbiased high-throughput RNA interference technology was used to screen cells that showed HBV inhibition after IFN-α treatment. Among 711 epigenetic modifiers, SET domain containing 2-mediated K525 STAT1 methylation is an important antiviral signaling mechanism (94). Activating the JAK signaling pathway further induces alternative signaling pathways such as mitogen-activated kinase-like protein, phosphatidylinositol 3-kinase, and nuclear factor Kappa-light chain enhancer of activated B cells (NF-κB), amplifying the strength and magnitude of type I IFN signaling (49). Though previous studies considered ISGs as IFN-induced protein-coding mRNAs, recent studies have shown that IFNs also mediate changes in the expression of many non-coding RNAs, including long non-coding RNAs and miRNAs (95).

The ISG gene pool is complex and large. Next-generation RNA sequencing studies have shown that IFNs regulate ~10% of all human genes. Moreover, studies comprehensively examining ISG expression in transcriptomes of different animals identified 62 core ISGs (96). Furthermore, several antiviral ISGs with critical roles have been discovered by identifying genes that are aberrantly expressed during viral infection inhibition. Among them, anti-myxovirus protein (MX)1 is the first classical effector molecule found to inhibit virus entry, primarily by preventing early-stage viral replication (97). In addition, interferon-induced transmembrane protein 3 (IFITM3) prevented the membrane fusion process of virus entry into cells *via* the endocytic pathway (98). Protein kinase R (PKR) and zinc antiviral protein are typical ISGs that inhibit viral protein production (99, 100). Therefore, different ISGs can block the HBV life cycle *via* corresponding pathways and play an essential role in regulating IFN-induced immune response and antiviral processes.

### The mechanism of ISGs in regulating HBV infection

IFN-α is an antiviral drug with a limited treatment course. It acts on important biological processes including HBV replication and transcription by enhancing immune cell function, increasing cytokine levels, inducing ISG expression, and activating multi-antiviral proteins *via* the IFN signaling pathway, thereby playing a dual role in immune and antiviral regulation (10). Various ISGs exert anti-HBV effects in the host *via* different mechanisms (**Table 2**). Host cells infected with viruses can immediately recognize their pathogen-associated molecular pattern, promoting the viability of B cells activated by transcription factors IFN regulator 3 or 7 and NF-κB. This process initiates the expression of the genes of type I IFNs and proinflammatory cytokines, inducing downstream ISGs to establish an antiviral host cell environment with antiviral effects (97).

**Table 2:** Summary of major anti-HBV ISGs.

ISGs	Characterization	Antiviral function	Mechanism	Different stages of HBV infection
**MX2 (** 101)	Myxovirus resistance (Mx) protein, an evolutionarily conserved dynein-like large GTPase	MX2 can inhibit HBV infection and proliferation by reducing cccDNA level and inhibiting HBV RNA transcription.	When pgRNA transcription is driven by HBV’s own promoter and enhancer from the add-on vector, MX2 reduces HBV DNA replication by downregulating all replication markers	Inhibits HBV cccDNA formation and RNA transcription
**SAMD4A (** 35)	SAMD4A is reported to be a mammalian homolog of *Drosophila* Smaug and to regulate post-transcriptional processes.	SAMD4A and its homolog SAMD4B can reduce HBV replication	SAMD4A mediates viral degradation by binding to the SRE site in viral RNA.	Promotes HBV RNA degradation and inhibits HBV replication
**IFI6 (** 102)	IFI6 belongs to the *FAM14* family localized on chromosome 1P35 and is an ISG	The overexpression of IFI6 inhibits HBV replication and translation in hepatocytes	IFI6 reduces HBV transcription and translation by inhibiting the ENHII/Cp promoter activity	Inhibits HBV DNA replication and RNA transcription
**TRIM14 (** 103)	The members of the TRIM family are known for their RING finger E3-ubiquitin ligase activity -including a RING domain, 1 or 2 b-box domains, and associated coiled-coil domains in the amino-terminal region	Type I IFN-stimulated gene TRIM14 controls HBV replication by targeting HBx	The TRIM14 SPRY domain interacts with the C-terminus of HBx, which may block the role of HBx in promoting HBV replication by inhibiting the formation of the SMC-HBX-DDB1 complex.	Inhibits HBV RNA transcription and HBV replication
**TRIM25 (** 104)		IL-27-dependent induction of TRIM25 inhibits HBV replication	Il-27 signaling is required for TRIM25 induction by type I IFN, and the transcription factors STAT1 and STAT3 play a role in TRIM25 induction.	Inhibits HBeAg secretion and HBV DNA replication
**ISG20 (** 105)	ISG20 has antiviral function against a variety of RNA viruses and is a 3’-5’ exonuclide induced by type I and type II IFNs.	ISG20 can bind and degrade HBV transcription factors and inhibit HBV replication.	ISG20 inhibits the HBV activity by binding to EnhII/Cp and inhibits HBV transcription by binding to YTHDF2 and recognizing m6A modifications.	Inhibits HBV transcription
**miR-122 (** 106)	MiR-122 is a mammalian liver-specific microRNA that is highly expressed in the liver, accounting for 70% of the total miRNA population in the liver.	MiR-122 significantly inhibited HBV expression and replication	MiRNA-122 was positively correlated with ADAR1 expression, and NT5C3 was identified as the miR-122 target.	Inhibits HBV DNA formation and RNA transcription
**ADAR1 (** 107)	ADAR1 is an ISG that catalyzes covalent modification of RNA substrates and produces inosine through C-6 deamination of hydrolyzed adenosine.	ADAR1 inhibited MAVS expression and reduced HBV marker levels *in vitro* and *in vivo*.	ADAR1 represses MAVS expression through human antigen R (HuR)-induced post-transcriptional regulation	Inhibits HBV DNA replication, RNA transcription, protein expression, and viral antigen packaging levels.
**IDO (** 108)	IDO is an IFN-γ-induced enzyme that catalyzes tryptophan degradation	IDO effectively reduced HBV DNA content in cells without affecting viral RNA stabilization.	IDO can inhibit viral genome replication and translation, and this antiviral effect is mediated by tryptophan deprivation.	Inhibits HBV DNA replication and protein translation
**OAS (** 109)	OAS encoded by the OAS gene uses adenosine triphosphate to synthesize 2’,5’ -oligadenylate (2’, 5’AS) in a 2’ -specific nucleotide transfer reaction, which activates latent ribonucrenase, leads to viral RNA degradation and inhibits viral replication	OAS gene variants may play an important role in the response to IFN-α	Polymorphism of IFN-induced gene OAS is associated with response to IFN-α therapy in chronic HBV infection	Promotes HBV RNA degradation and inhibits HBV replication

IFN-α-induced ISG MX2 reduces HBV cccDNA expression by inhibiting viral RNA synthesis, an important anti-HBV function. MX2 represents a novel HBV inhibitor with therapeutic potential (101). APOBEC3G is an IFN-α-induced cytosine deaminase that deaminates cytosine to uracil in single-stranded DNA replication, inhibiting the coding and replication ability of HBV (110). In a study, cell-based assays were performed to screen 285 human ISGs to check their anti-HBV activity, finding *SAMD4A* to be an important anti-HBV ISG and a strong repressor of HBV replication. It can be used in IFN-HBV treatment. *SAMD4A*/*B* expression was associated with human HBV sensitivity (35). In addition, IFN-α-inducible protein 6 (IFI6) inhibited HBV replication in cell and mouse model by reducing the expression of the gene of HBV enhancer II and core promoter (EnhII/Cp); thus, increasing *IFI6* expression may be a potential therapeutic approach for inhibiting HBV infection (102).

Another study showed that the SPRY domain of tripartite motif containing 14 (TRIM14) interacted with the C-terminus of the HBV X protein (HBx) and might block HBV replication by inhibiting the formation of the structural maintenance of chromosome protein (SMC)-HBx- DNA damage-binding protein 1 (DDB1) complex (103). Other studies have shown that the IFN-interleukin (IL)-27-TRIM25 signaling pathway is induced by type I IFNs and inhibits HBV replication, identifying the ISG *TRIM25* as a potential therapeutic target for HBV infection (104). In addition, *TRIM5γ* and *TRIM31* were identified as key genes interacting with HBx that promote its degradation among the 145 ISGs examined, identifying them as potential therapeutic strategies for IFN-resistant patients with HBV infection (111). ISG20 is a 3′-5′ exonuclease that binds and degrades HBV transcripts (105). *ISG20* is primarily induced by IFN-β, reducing HBV gene expression and inhibiting HBV enhancer activity by binding to EnhII/Cp regions (112). Moreover, m6A reader protein YTH domain family 2 (YTHDF2) regulates *ISG20* expression by selectively recognizing and processing N6-methyladenosine (m6A)-modified HBV transcripts for degradation (105).

Moreover, studies have shown that IFN-α treatment significantly decreases microRNA-122 (miR-122) expression in hepatocytes, targeting ISG 5′-nucleotidase, cytosolic III (NT5C3), an inhibitor of miR-122 expression, and potentially inhibiting IFN-α function in HBV treatment (106). Other studies have shown that hepatocyte-specific miR-122 expression positively correlates with adenosine deaminase acting on RNA gene (*ADAR1*) expression. Exogenous miR-122 reduces HBV RNA and DNA, and p53 is also involved in the ADAR1-mediated reduction of HBV RNA (113). In addition, studies have shown that IFN-α attenuates mitochondrial signaling protein (MAVS) by RNA editing, which is mediated by ADAR1 antiviral therapy. These results indicate that combining MAVS with IFN-α has potential clinical applications in the studies on HBV infection (107).

ISG stimulator of interferon response the cyclic guanosine monophosphate-adenosine monophosphate (cGAMP) interactor (STING) is an important DNA-mediated regulator regulating the natural immune response of the body and a potential therapeutic target in HBV infection (114). Studies have shown that activation of STING signaling pathway can effectively reduce the severity of liver injury in chronic HBV mouse models, which may be a promising approach to prevent HBV virus proliferation and HBV-related liver fibrosis (115, 116). Furthermore, IFN-α reduces HBV cccDNA content by regulating the general control non-repressed 5 protein-mediated succinylation of histone H3K79 in HBV-infected human liver-chimeric mice. Therefore, IFN-α can inhibit HBV transcription at the epigenetic level (117). Indoleamine 2, 3-dioxygenase (*IDO*) is an ISG that can effectively reduce intracellular HBV DNA levels and the main IFN-γ regulatory gene in hepatocytes to produce an anti-HBV response (108). Moreover, the downstream signaling pathway of IFN-λ was identified by a proteomic method. *IFITM3*, 5′-3′ exoribonuclease 2 *(XRN2)*, and 5’-nucleotidase, cytosolic IIIA (*NT5C3A)* expression were upregulated, and ISG transcription was activated to inhibit HBV replication (118).

In addition, ISG expression as a predictor of clinical efficacy is also an attractive strategy. Single nucleotide polymorphisms (SNPs) in the 2′,5′-oligoadenylate synthetase gene (*OAS*) in patients play a major role in predicting the efficacy of IFN treatment against CHB (119). Additionally, SNPs in *IL28B* and *OAS* were correlated with the clinical efficacy of IFN therapy in children with CHB, suggesting that they might be a new important consideration in treating CHB with IFNs (120).

According to recent studies, ISGs may also be involved in the mechanism by which HBV antagonizes IFNs and inhibits IFN efficacy. Studies have shown that IFN-α treatment activates STAT1 nuclear translocation and ISG expression. Therefore, HBV inhibits STAT1 nuclear translocation and interferes with ISG transcription in hepatocytes, blocking IFN-α signaling and causing a poor treatment response (71). In addition, HBV has molecular mechanisms that promote resistance to IFN therapy. HBV infection increases HBV polymerase levels and inhibits ISG induction, resulting in the poor antiviral efficacy of IFN-α in HBV mouse model (121). In addition, HBV precore protein P22 can reduce ISG expression and IFN-stimulated response element activity and inhibit IFN-α signaling by blocking the JAK/STAT signaling pathway and STAT nuclear translocation (122). Spliceosome-associated factor 1 can reduce the antiviral activity of IFN-α by attenuating JAK/STAT signaling and reducing the expression of ISGs such as *MX*, *OAS*, and *PKR* in HepG2 cells (123).

**Figure 3:**
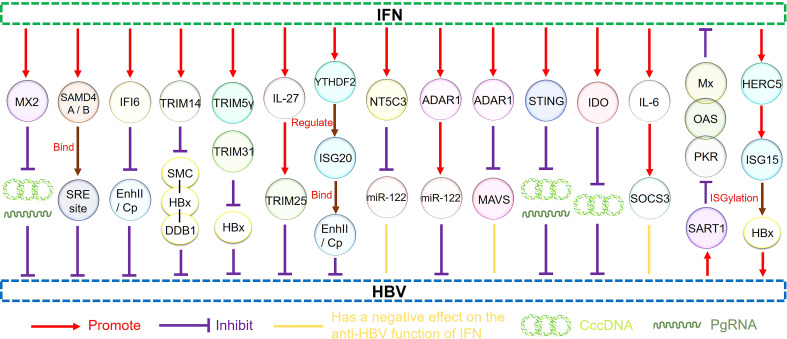
The regulatory pathways of ISGs.

Moreover, *IL-6* expression impaired the efficiency of IFN-α-mediated HBV suppression in hepatocytes by upregulating the suppressor of the cytokine signaling 3 genes (*SOCS3*). Therefore, *SOCS3* downregulation can improve the antiviral activity of IFNs in HBV-replicating hepatocytes to a certain extent, representing a novel therapeutic strategy that may effectively target HBV infection (124). Other studies have shown that HBV can promote miR-146a transcription, inhibiting STAT1 and leading to IFN resistance. Therefore, this mechanism represents a promising research target for recovering the effects of IFN-α in HBV treatment (125). Moreover, the homologous to the E6-AP carboxyl terminus and RLD domain containing E3 ubiquitin-protein ligase 5-mediated modification of HBx by ISG15 increased HBV replication, resulting in HBV resistance to IFN-α therapy (126). Understanding the interaction between HBV and ISGs and ISG regulation by HBV to produce IFN antagonism will be helpful for further anti-HBV research (**Figure 3**).

## Discussion

As CHB can lead to immune impairment and tolerance, immunomodulatory IFN therapy offers particular mechanistic advantages in antiviral regulation than NAs, which cannot directly target the viral cccDNA reservoir (10). Studies have shown that IFN-α treatment can promote the degradation of HBV pgRNA in transgenic mice and induce the epigenetic inhibition of cccDNA in human hepatocytes both *in vitro* and *in vitro* (127, 128). The PEGylated form of IFNs is an immunomodulator providing the highest functional cure rate over a fixed treatment period (129). However, IFN therapy also has certain disadvantages. Loss of HBsAg associated with HBV DNA suppression is a desirable outcome of antiviral therapy. However, only 3%–11% of patients benefit from IFN therapy, and most need to continue drug therapy indefinitely (130). PEG-IFN-α is effective in only ~20% of patients, and its use is limited by its side effects. Therefore, developing new therapies that can be used in limited therapeutic courses to cure HBV infection is imperative (131). IFN therapy requires new drug combination strategies, IFN optimization, and more reliable biomarkers for clinical diagnosis. New IFN subtypes and delivery methods can be explored to improve the clinical effect of IFN treatment. Besides, with the development of animal models, more and more HBV animal models such as mice, chimpanzees, ducks, woodchucks and monkeys have been used to study the mechanism of IFN regulation of ISG, which helps us to further understand the method of suppressing HBV *in vivo*. Moreover, IFN-induced ISGs also play an important role in HBV progression. An important research direction might be to improve the efficacy of IFN treatment by ISGs targeting HBV.

Studies have shown that IFNs can achieve its powerful antiviral performance by inducing ISGs, regulating the immune response of the body, and acting directly on the enhancer and promoter sequences of infected viruses (97). Many ISGs are upregulated by IFN signaling and target different phases of the HBV life cycle (132). ISGs can act as effectors produced by IFN stimulation to exert a direct antiviral effect. The overexpression of ISGs that inhibit HBV HBeAg expression, including SAMD4A, MX2, IFI6, TRIM family members, ISG20, miR-122, ADAR1, and IDO, is conducive to the use of IFNs in HBV treatment. Other studies have shown that ISGs such as MAVS, NT5C3, and SOCS3 attenuate the anti-HBV effect of IFNs, and the downregulation of their expression may be an effective treatment strategy.

In addition, the efficacy of IFN treatment against CHB varies greatly among patients. Previous studies have shown that ISGs may be related to the outcome and antiviral efficacy after HBV infection, making them promising biomarkers for predicting the clinical efficacy of IFN treatment (120). However, HBV can also regulate ISGs to inhibit IFN signal transduction and promote viral proliferation. Hence, the mechanism of HBV acting on ISGs can also be used as a breakthrough point for treatment (122). Moreover, some important ISGs may contribute to the development of adjuvants for viral vaccines. IDO expression is increased in hemodialysis patients and affects the immune response to HBV vaccination (133). In addition, the induction of humoral and cellular immune responses to HBV vaccine can be upregulated by the STING ligand cGAMP (134). Studies have shown that ISG15 plays a critical role in MDA5-mediated antiviral response, and this mechanism may facilitate the development of new antiviral drugs and vaccines against COVID-19 (135). Besides, toll-like receptor (TLR) has been shown to control ISG mRNA levels, and a variety of vaccines with TLR as adjuvants have been shown to be effective in preclinical studies (136). It has also been demonstrated that the regulation of constitutive ISGs in tumor cells contributes to the enhancement of the antitumor response to Newcastle disease virus-infected tumor vaccines (137). Therefore, the regulatory mechanism of ISGs is a promising direction for the research of HBV vaccine adjuvants. IFNs can induce many ISGs. At present, only a few ISGs are associated with the antiviral activity of HBV, and the related biology and antiviral action mechanism of most remaining ISGs still need to be explored in depth. In addition, no unified model for predicting the efficacy of IFN treatment on CHB is available, and studies on the predictive efficacy of ISGs are limited. Further basic and clinical studies are needed to identify the target and mechanism of IFNs in HBV treatment by the combined effect of IFNs and ISG regulation, which may be a more promising strategy for clinical research to cure HBV.

## Conclusions

HBV treatment remains an important medical problem. IFNs are commonly used immunomodulatory agent that suppresses HBV. The inhibitory mechanism of IFNs on HBV is complex and includes regulating ISG to inhibit HBV, which has received much attention. IFNs induces various ISGs to reduce HBV transcription, replication, and translation. Understanding the mechanism of ISG regulation of HBV will help identify new targets that promote the therapeutic effect of IFNs and develop new clinical strategies for HBV treatment.

## Author contributions

Conceptualization, QL, YG and DW. Funding acquisition, DW. Project administration, DW. Supervision, DW. Writing – original draft, QL. Writing – review & editing, QL, BS, YZ, ZJ, RL, CL, YJ, YG and DW. All authors contributed to the article and approved the submitted version.

## Funding

This work was supported by the Jilin Province Science and Technology Development Program under Grant 20210204013YY, the National Natural Science Foundation of China under Grant 82003985, the Foundation of Jilin Educational Committee under Grant JJKH20210995KJ, the Jilin Scientific and Technological Development Program under Grant 20220505033ZP, and the Grain, Oil and Food Deep Processing Scientific Research Project of Key Laboratories of Colleges and Universities in Jilin Province under Grant [2019] No. 004.

## Conflict of interest

The authors declare that the research was conducted in the absence of any commercial or financial relationships that could be construed as a potential conflict of interest.

## Publisher’s note

All claims expressed in this article are solely those of the authors and do not necessarily represent those of their affiliated organizations, or those of the publisher, the editors and the reviewers. Any product that may be evaluated in this article, or claim that may be made by its manufacturer, is not guaranteed or endorsed by the publisher.
